# ​​Trends in self-reported headache disorder in Spain between 2006 and 2023: the role of sociodemographic factors

**DOI:** 10.1186/s12889-026-26744-3

**Published:** 2026-02-27

**Authors:** Miguel Á. Huerta, Jose A. Moral-Munoz, Masahito Katsuki, Yasuhiko Matsumori, Alejandro Salazar

**Affiliations:** 1 Department of Pharmacology and Neurosciences Institute (Biomedical Research Center), University of Granada, Granada, Spain; 2 Biosanitary Research Institute ibs.GRANADA, Granada, Spain; 3 Department of Pharmacology, University of Cambridge, Cambridge, United Kingdom; 4 Department of Nursing and Physiotherapy, University of Cadiz, Cadiz, Spain; 5 Biomedical Research and Innovation Institute of Cadiz (INiBICA), Cadiz, Spain; 6 Observatory of Pain, Grünenthal Foundation-University of Cadiz, Cadiz, Spain; 7 Insight Science Foundation Ireland Research Centre for Data Analytics, School of Human and Health Performance, Dublin City University, Dublin, Ireland; 8 Physical Education and Health Center, Nagaoka University of Technology, Niigata, Japan; 9 Sendai Headache and Neurology Clinic, Sendai, Miyagi Japan; 10 Department of Statistics and Operational Research, University of Cadiz, Cadiz, Spain

**Keywords:** Pain, Migraine, Chronic disease, Epidemiology, Europe

## Abstract

**Introduction:**

Headache conditions impose a significant global socioeconomic burden; however, long-term trends in their prevalence remain underexplored. This study aimed to analyze the evolution in self-reported headache disorder (SRHD) in Spain from 2006 to 2023, focusing on its relationship with demographic factors (sex, age, and social class) and the potential impact of the COVID-19 pandemic.

**Methods:**

A repeated cross-sectional study using seven national health surveys in Spain (2006–2023; 37,429 − 41,929 participants) analyzed SRHD adjusted by sex, age, social class, and education. Trends were tested with the Mann–Kendall method.

**Results:**

SRHD prevalence declined from 14.5% in 2006 to 10.8% in 2023, showing a negative, non-significant trend (total τ = − 0.62, *p* = 0.07; physician-diagnosed τ = − 0.33, *p* = 0.38). The reduction was more evident among women, older adults, and individuals from lower socioeconomic groups. Prevalence was approximately 2.5-fold higher in women, peaked between 25 and 54 years, was lowest in the youngest (15–24) and oldest (> 75) groups, and decreased with higher social class and education. The 2020 data point, collected during the COVID-19 pandemic, deviated from the trend but was not a statistical outlier, and its exclusion reduced the magnitude of the downward slope without altering its non-significant nature.

**Conclusion:**

SRHD prevalence showed a non-significant decline over the past 17 years in Spain. The reduction was more evident among women, older adults, and lower socioeconomic groups, indicating that demographic and social determinants may shape these temporal dynamics.

**Supplementary Information:**

The online version contains supplementary material available at 10.1186/s12889-026-26744-3.

## Introduction

Headache disorders impose a high socioeconomic burden worldwide that drastically affects the quality of life of patients and their families [1]. Migraine is the second most common cause of disability among young women [2], whereas tension-type headache (TTH) is the most prevalent primary headache disorder and contributes significantly to population-level morbidity [3, 4]. Despite the high disease-associated disability, migraine is often underdiagnosed [5–7], partly because patients do not consult physicians [8–10]. It is also associated with a wide range of risk factors, triggers, and comorbidities [11]. The estimated annual costs per patient are around $9000 in the US [12], and the costs of the pharmacological treatment are drastically increasing as several life-changing therapies have recently emerged: monoclonal antibodies, gepants, among others [13].

The current global migraine prevalence is 14–15%, which accounts for 4.9% of global ill health, quantified as years lived with disability [7]. However, the incidence in Europe is slightly lower than globally, according to the Global Burden of Disease (GBD) [14]. A large systematic review that meta-analyzed data from 302 studies (involving more than 6 million participants) reported a higher prevalence in South America and Europe [15]. Specifically, the prevalence was estimated to be approximately 9–15% in Spain [6, 16, 17], affecting more than 4.5 million people [16]. In contrast, TTH is more prevalent than migraine, with annual prevalence estimates around 20–30% in adults [18].

Despite the high burden of these conditions and the large number of people affected, most studies have reported only cross-sectional prevalence estimates (typically at a single time point, often within a one-year period), without examining trends over time [6, 15]. The most recent study to report long-term trends is the Global Burden of Disease (GBD) study, which found that the global incidence of migraine reached 87.6 million in 2019, an overall increase of 40.1% compared to 1990 [14]. However, this rise is primarily attributed to global population growth, as the relative prevalence of migraine and TTH has actually declined worldwide by 1.8% and 7.3%, respectively [19]. Temporal variation in prevalence values differed across regions, with only about one-third of countries experiencing an increase. These increases were closely linked to national economic status—for example, countries with the lowest Sustainable Development Index, such as Equatorial Guinea, showed the most significant rises [14]. Conversely, the decline in the incidence of migraine and TTH was more consistent across Europe, with a particularly notable reduction observed in Spain [14]. Similarly, a downward trend in the prevalence of other pain-related conditions has been observed in Spain over the past two decades [20].

To the best of our knowledge, no studies have evaluated the evolution of self-reported headache disorder (SRHD) over the years by extracting data from different time points (comparing surveys with equivalent methodology and populations) and analyzing the association of the variation with other sociodemographic variables that may influence it. Accordingly, the objective of the present study was to analyze the evolution of SRHD in the general Spanish population (aged ≥ 15 years) from 2006 to 2023 by extracting data from seven equivalent surveys. In addition, the relationship between the variation and other demographic variables (sex, age group, social class, and educational level) was analyzed. The results obtained are intended to be the basis of healthcare strategies and allocation of resources within the healthcare system. In addition, they will stimulate research into the underlying causes of the trend, so that more effective preventive measures can be implemented.

## Materials and methods

### Study design

This is an ecological time-series analysis based on population-level prevalence estimates obtained from the National Statistics Institute of Spain (INE, in the Spanish acronym) (Instituto Nacional de Estadística). The study followed the Strengthening the Reporting of Observational Studies in Epidemiology (STROBE) guidelines [21].

### Data source

**Fig. 1:**
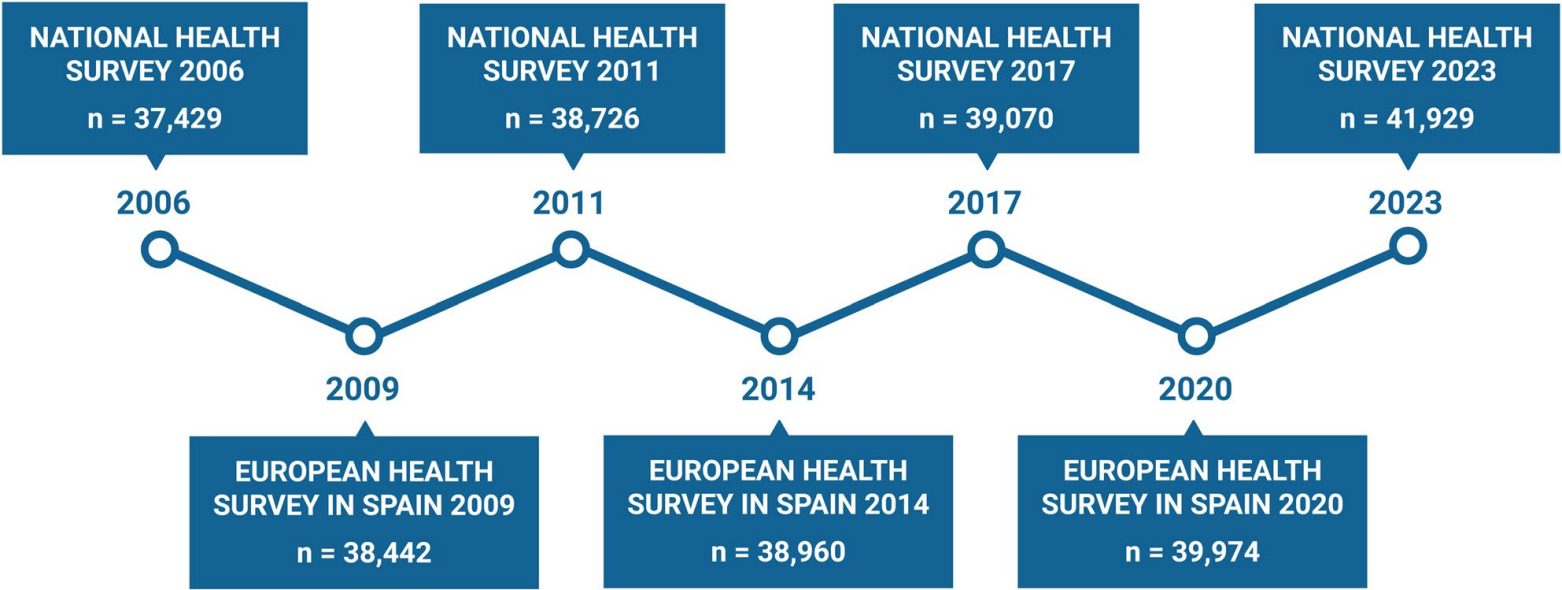
Timeline of the surveys and number of participants included

Data was collected from seven surveys conducted between 2006 and 2023 in the Spanish general population, with samples ranging from 37,429 to 41,929 subjects per survey. Specifically, as shown in Fig. 1, four of these (2006, 2011, 2017, and 2023) were the National Health Survey (NHS), and the others (2009, 2014, and 2020) were the European Health Survey in Spain (EHSS). All surveys were conducted by the INE, which is associated with the Spanish Ministry of Health and Social Services. Other information about the methodology of the surveys can be found on the INE-specific web page for the National Health Survey (2006, 2011, 2017, and 2023) and the European Health Survey in Spain (2009, 2014, and 2020).

### Study population and definition of the evaluated condition

The study population was representative of the general Spanish population (aged ≥ 15 years). It was selected using a probabilistic multistage sampling method, with the first-stage units being census sections and the second-stage units being the main family dwellings. The presence of SRHD was considered for individuals who responded affirmatively to the question: “Have you suffered from migraine or frequent headache in the last 12 months?”. Additionally, an individual was considered to have been physician-diagnosed with SRHD if they responded affirmatively to the following question: “Has the doctor told you that you suffer from migraine or frequent headache?”.

### Variables included in the analysis

In addition to the presence of SRHD and physician-diagnosed SRHD, we also collected the following information:

#### Sex

Dichotomous variable (man or woman).

#### Age

The following age groups: 15–24, 25–34, 35–44, 45–54, 55–64, 65–74, and > 75 years.

#### Social class

Social class categories were assigned according to occupation:

- GROUP I: Directors and managers of companies with 10 or more employees and professionals traditionally associated with university degrees.
- GROUP II: Directors and managers of companies with fewer than 10 employees, professionals traditionally associated with university degrees, and other technical support professionals. Athletes and artists.
- GROUP III: Intermediate occupations (administrative employees and professionals supporting administrative management and other services) and self-employed workers.
- GROUP IV: Supervisors and workers in qualified technical occupations.
- GROUP V: Qualified workers in the primary sector and other semi-qualified workers.
- GROUP VI: Unqualified workers.

#### Educational level

Educational level was categorized according to the classifications provided by the INE in the health surveys. Respondents were grouped based on their highest completed level of formal education. For analytical purposes, we grouped educational attainment into three categories:

1. Lower/basic education: completed at most primary education, including those without formal studies or with incomplete compulsory education.
2. Intermediate education: completed secondary education (e.g., compulsory secondary education, high school, vocational training of intermediate level).
3. Higher education: completed university-level studies or higher vocational training (higher vocational training, university degrees, or postgraduate education).

### Data extraction

The numerical data for this study were directly obtained from each survey available in the INE online database by MAH, ensuring precise extraction of all relevant information. The INE provides global data in tabulated formats and data segmented by subgroups of interest. These data are derived from National Health Surveys using probabilistic multistage sampling designs. It is worth mentioning that the prevalences used in this study are already weighted and calibrated by the INE to account for the complex survey design and to ensure national representativeness. As no individual-level microdata were analyzed, and no prevalence estimates were calculated by the authors, the application of survey weights or design-based variance estimation was unnecessary. This procedure was repeated for each survey, resulting in a comprehensive dataset encompassing all necessary information. To ensure data accuracy and minimize errors, AS and JAMM independently cross-checked the extracted data. This verification process involved confirming consistency in values, proper alignment with corresponding variables, and compliance with the established methodology. Any discrepancies identified were addressed and resolved collaboratively, guaranteeing a robust and reliable final dataset for analysis.

### Statistical analysis

Data were analyzed using GraphPad Prism software (v8.0; GraphPad Software, Inc., USA). For each survey, the prevalence of SRHD had been calculated by the INE as the number of cases divided by the total population, after applying their adjustment described in the survey methodology. Prevalence was also specifically determined by sex, age group, and social class. The results are shown as line and bar plots, where the x-axis represents time and the y-axis represents prevalence. A time-series analysis was performed for the evolution of the total and reported SRHD prevalence over time. The Mann-Kendall test was used to assess the significance of a potential trend in the data. To evaluate the potential impact of the COVID-19 pandemic, a Grubbs’ test was applied to determine whether the 2020 datapoint represented a statistical outlier. In addition, a standardized residual analysis based on a linear model excluding 2020 was conducted.

## Results

### Overall prevalence of self-reported headache disorder between 2006 and 2023

**Fig. 2:**
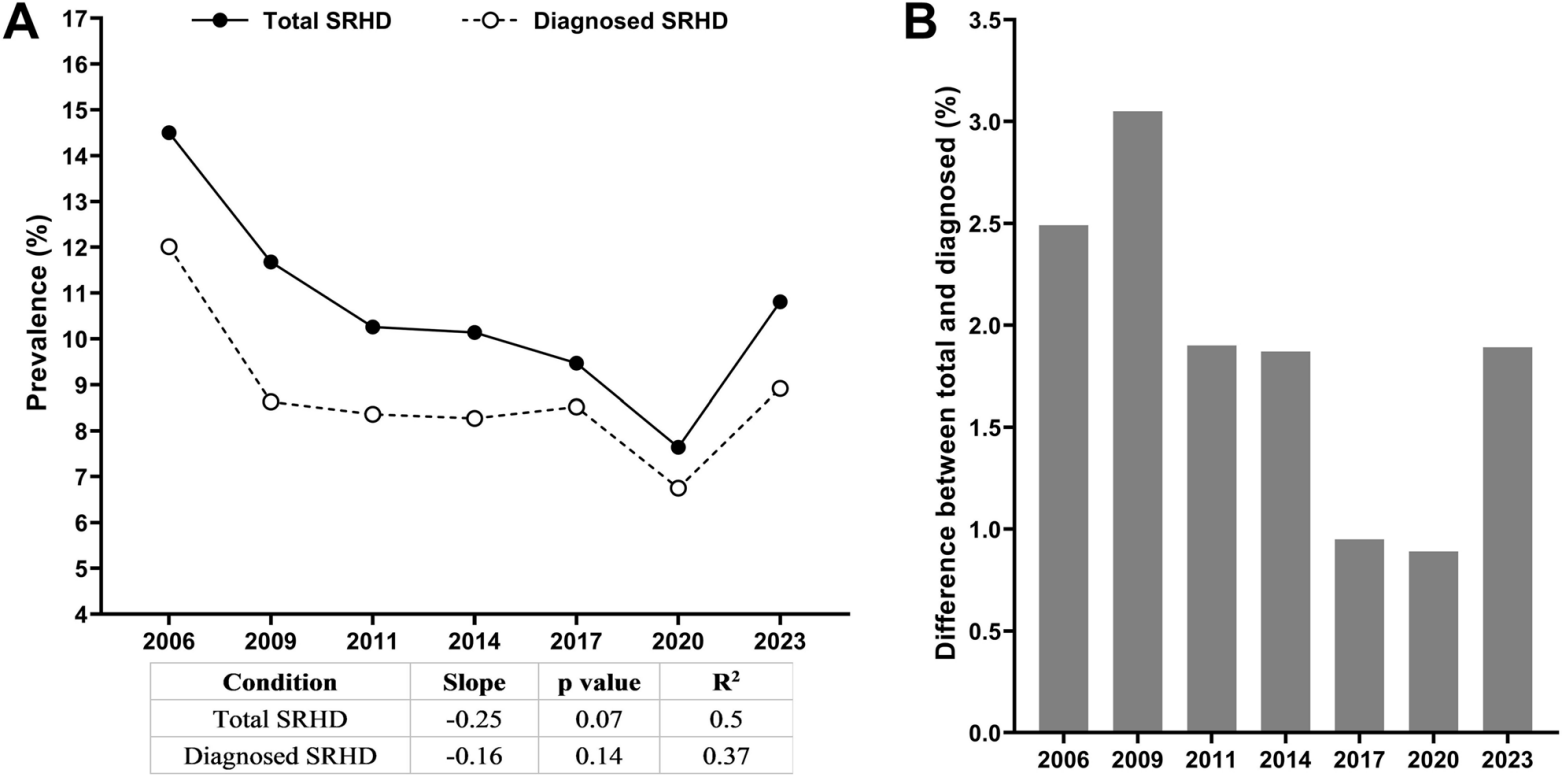
Prevalence of total and physician-diagnosed self-reported headache disorder between 2006 and 2023. **a** Temporal trends in total prevalence (solid line) and physician-diagnosed prevalence (dashed line), with linear regression statistics. **b** Absolute difference in prevalence (%) between total and physician-diagnosed values for each survey year, illustrating the reduction in the diagnostic gap over time

A descending slope over time was observed for the prevalences of SRHD (total SRHD: slope = -0.25 and R^2^ = 0.50; physician-diagnosed SRHD: slope = -0.16 and R^2^ = 0.37) (Fig. 2A). The maximum prevalence (14.50%) was obtained in 2006, and the minimum (7.64%) in 2020. When comparing total SRHD prevalence to physician-diagnosed cases, clear differences emerged: as expected, reported prevalence rates were higher than those confirmed by diagnosis. However, a slight reduction in the gap between reported and physician-diagnosed cases was observed over time, particularly in 2017 and 2020 (Fig. 2B).

### Prevalence of self-reported headache disorder stratified by sex between 2006 and 2023

**Fig. 3:**
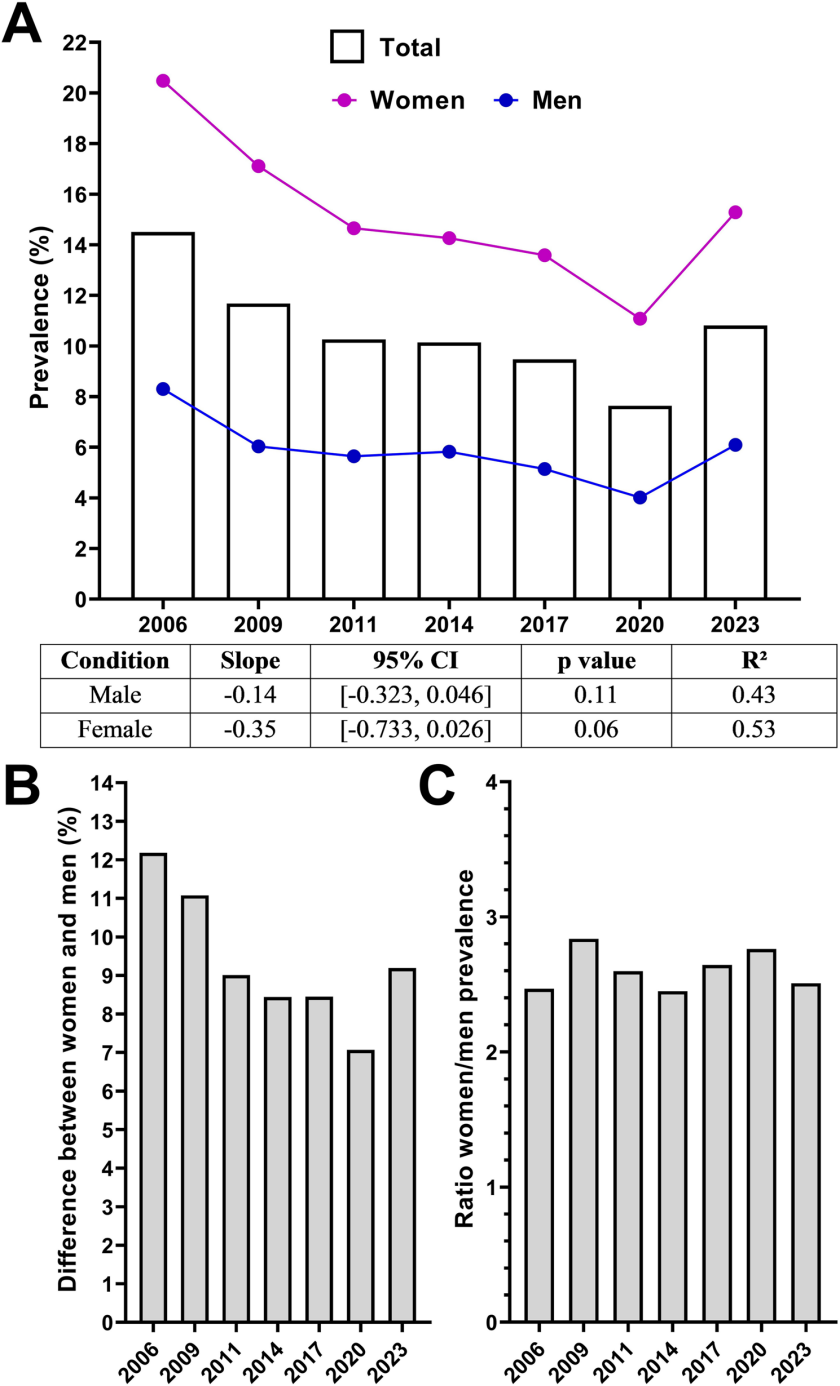
Prevalence of self-reported headache disorder stratified by sex between 2006 and 2023. **a** Temporal trends in prevalence for the total population (bars), women (magenta line), and men (blue line), with linear regression parameters and statistical results for each sex. **b** Absolute difference in prevalence (%) between women and men for each survey year. **c** Ratio of prevalence in women compared to men (women-to-men ratio) for each year

The analysis stratified by sex showed clear differences, with women (purple line) presenting significantly higher values (approximately 2.5 times) than men (blue line) (Fig. 3A). Although the prevalence decreased in both sexes, the reduction was more marked in women, with a steeper decline (-0.35 vs. -0.14 for men), yet it did not reach statistical significance in either case (*p* = 0.06 for women; *p* = 0.11 for men). This was associated with a decrease in the difference between sexes, which decreased from 12.18% in 2006 to 9.19% in 2023 (Fig. 3B), although the ratio of women/men remained stable in the range of 2.45–2.83 during the whole period (Fig. 3C).

### Prevalence of self-reported headache disorder stratified by age groups between 2006 and 2023

**Fig. 4:**
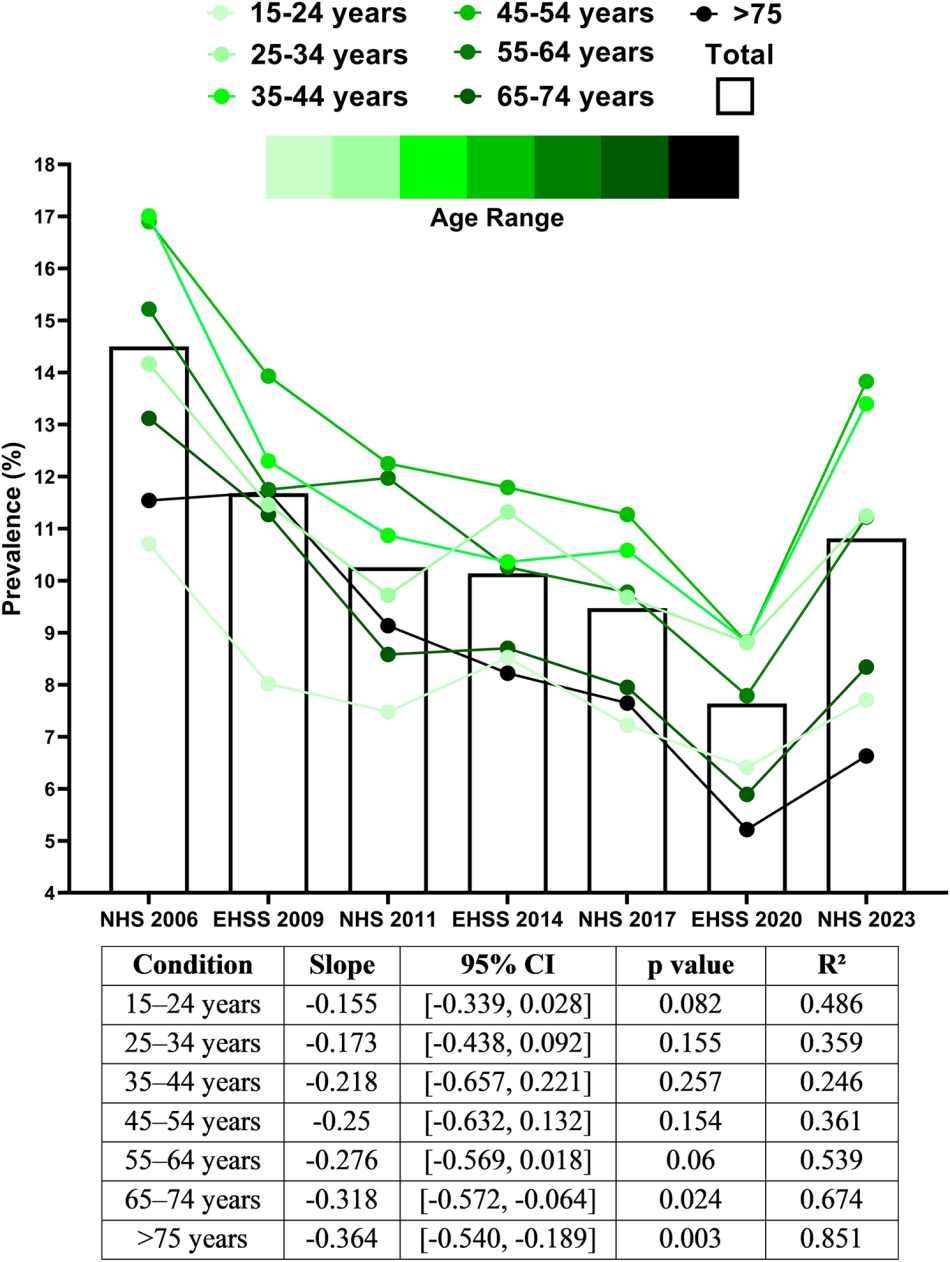
Prevalence of self-reported headache disorder stratified by age groups between 2006 and 2023

SRHD prevalence was clearly influenced by age, and its temporal evolution differed slightly across age groups (Fig. 4). Middle-aged groups (35–44, 45–54, and 55–64 years) showed a slight, non-significant decrease in prevalence, closely resembling the trend observed in the overall population (shown in grey bars). Younger groups (15–24 and 25–34 years) exhibited an even milder and non-significant decline. In contrast, older age groups (65–74 and > 75 years) showed a more pronounced decrease, which reached statistical significance (*p* < 0.05). Interestingly, SRHD prevalence was higher in the intermediate age groups (25–34, 35–44, and 45–54 years) and lower in the extreme age groups (15–24, 65–74, and > 75 years). In fact, the highest values were always observed in groups 25–34, 35–44, and 45–54 years, with values ranging from 17.01% in 2006 to 13.83% in 2023. Conversely, the lowest values were found in the 15–24 and > 75 years age groups, with values ranging from 10.71% in 2006 to 6.63% in 2023.

### Prevalence of self-reported headache disorder stratified by social class and educational level between 2006 and 2023

**Fig. 5:**
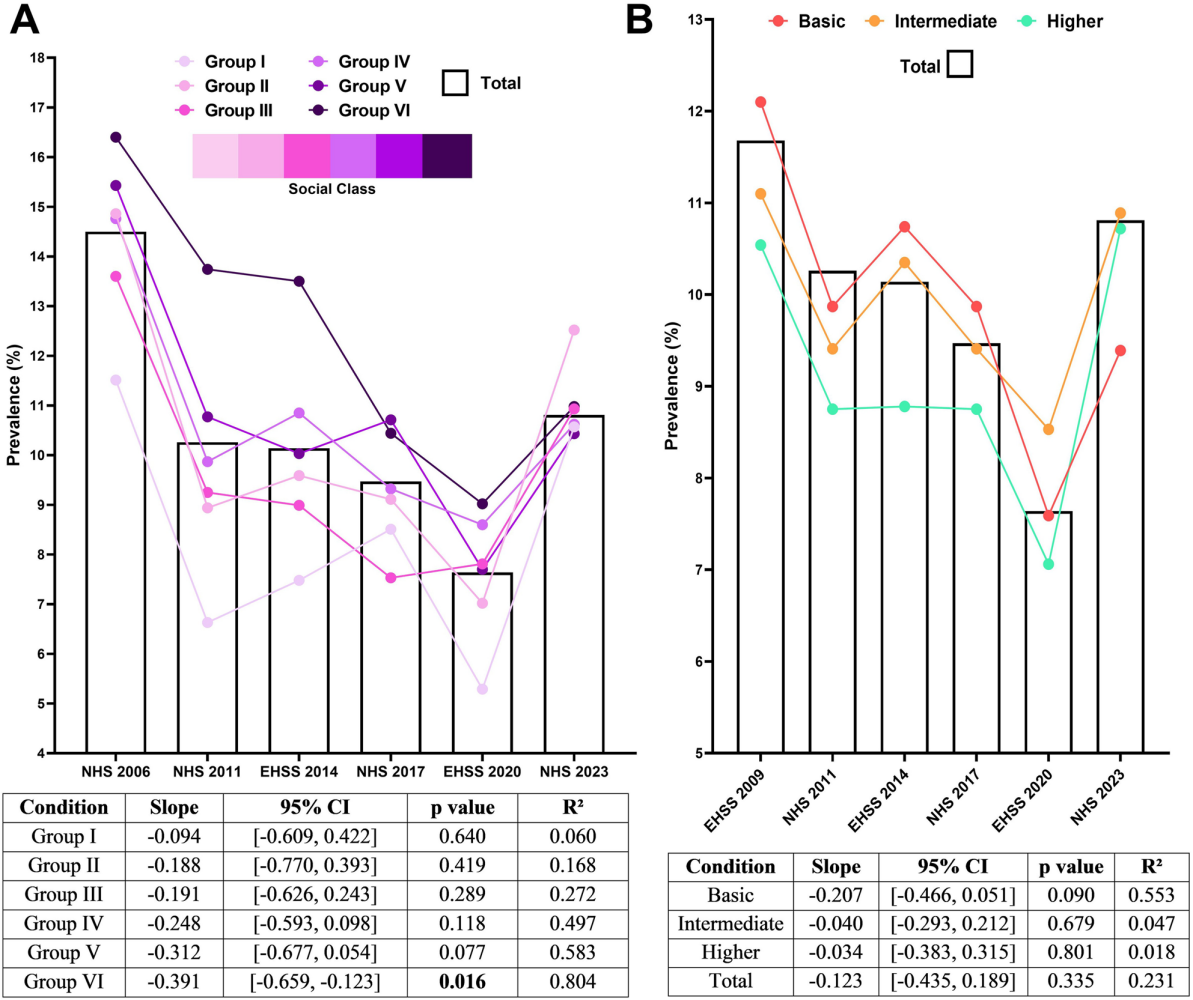
Prevalence of self-reported headache disorder between 2006 and 2023, stratified by sociodemographic factors. **a** Temporal trends by social class groups, with regression statistics shown in the accompanying table. **b** Prevalence by educational level group (basic, intermediate, higher) across survey years

Social class exerted a strong influence on SRHD prevalence, with higher values consistently observed among individuals from lower social classes, except in 2023, when prevalence rates were surprisingly similar across all groups (Fig. 5A). Accordingly, the highest prevalence values were found in Group VI (ranging from 16.40% in 2006 to 10.98% in 2023), while the lowest were observed in Group I (from 11.51% in 2006 to 10.57% in 2023). However, the temporal decrease in prevalence differed across social class groups. Specifically, higher-class groups (Groups I–III) exhibited smaller descending slopes (ranging from − 0.09 to − 0.19), none of which reached statistical significance (*p* > 0.05). In contrast, lower-class groups (Groups IV–VI) showed steeper declines (from − 0.25 to − 0.39), some of which achieved statistical significance (*p* < 0.05).

Similarly, SRHD prevalence was influenced by educational level, with higher prevalence observed among individuals with lower levels of education (Fig. 5B). Once again, the 2023 datapoint was an exception, showing an inverted pattern compared with previous years. A temporal decline in prevalence was detected only among individuals with basic education (slope = − 0.21; *p* = 0.09), whereas intermediate and higher educational levels showed no significant changes over time (slopes between − 0.03 and − 0.04; *p* > 0.05).

Overall, these analyses indicate that SRHD prevalence is shaped by both social class and educational attainment. Specifically, the higher the social class or educational level, the lower the prevalence. Moreover, a temporal decrease was observed exclusively among individuals from the lower social and educational strata.

### Time series analysis

A Mann–Kendall trend test was performed to evaluate the temporal evolution of SRHD prevalence between 2006 and 2023. The analysis revealed a negative, non-significant trend for both total (τ = − 0.62; *p* = 0.07) and physician-diagnosed prevalence (τ = − 0.33; *p* = 0.38), indicating mild but not statistically confirmed decreases over time in both datasets (Table 1). This result aligns with the general pattern observed in the linear regression, suggesting a modest downward tendency in prevalence that did not reach conventional levels of statistical significance.

**Table 1:** Tendency for the prevalence of self-reported headache disorder

Variable	M-K τ*	M-K p*
Total SRHD	-0.62	0.07
Physician-diagnosed SRHD	-0.33	0.381

*Abbreviations*: *SRHD* Self-reported headache disorder

*Kendall’s tau and *p*-value associated with the Mann-Kendall test for the significance of the trend over time

### Influence of COVID-19

A Grubbs’ test was conducted to assess whether the 2020 data point, collected during the COVID-19 pandemic, could be considered a statistical outlier within the temporal series. The result indicated that the 2020 value did not reach the threshold for statistical significance (G = 1.42, G₍crit₎ = 2.02, *p* > 0.05). Similarly, a standardized residual analysis based on a linear model excluding 2020 revealed a deviation of moderate magnitude (*r* = − 1.44), below the conventional cutoff for outlier detection (|r| > 2). Therefore, although the 2020 estimate deviated from the expected linear pattern, it cannot be classified as a statistical outlier.

To further evaluate the influence of this pandemic year on temporal trend estimates, a sensitivity analysis was performed excluding the 2020 survey wave (Figure S1). After exclusion, the Mann–Kendall test continued to indicate a non-significant negative trend (τ = −0.60, *p* = 0.136), and the slope of the linear regression was attenuated (β = −0.19% per year, *p* = 0.175), while maintaining a negative direction. These results indicate that the magnitude of the observed downward pattern is sensitive to the inclusion of the 2020 data point, although statistical significance is not reached in either scenario.

Taken together, these findings suggest that the lower prevalence estimate observed in 2020 likely reflects the exceptional social, behavioral, and healthcare-related circumstances associated with the COVID-19 pandemic rather than a random fluctuation or a true epidemiological shift.

## Discussion

This study analyzed data from seven national health surveys conducted in the Spanish general population (≥ 15 years) between 2006 and 2023, revealing a non-significant downward pattern in the prevalence of SRHD (from 14.50% in 2006 to 10.81% in 2023). The analysis of covariates (sex, age groups, social class, and educational level) showed that this decrease was more pronounced, and in some cases statistically significant, among women, individuals aged over 55 years, and those belonging to lower social classes or with lower educational levels. These subgroup-specific patterns suggest that the potential reduction in SRHD prevalence was not uniformly distributed across the population but rather concentrated in certain demographic and socioeconomic groups. The underlying causes of this pattern remain uncertain but are likely multifactorial, involving both biological and social determinants.

In line with our findings, the GBD Study 2017 (one of the most comprehensive studies to date due to its large sample size and global coverage) reported a slight decrease (1.8%) in the global prevalence of migraine between 1990 and 2016 [19, 22, 23], with similar reductions observed in Western Europe (1.2%), Spain (1.7%) [19], or stabilization in the USA [24]. The decline in TTH was even more pronounced, with a 5.7% decrease in Europe and a 6.5% decrease in Spain [19]. These findings suggest that, although prevalence estimates vary across regions and headache subtypes, downward or stable patterns have been reported in some large-scale epidemiological studies. Our results are broadly consistent with this literature in terms of direction and magnitude.

In view of our results, it is important to emphasize that the combined variable of SRHD aggregates migraine and other frequent headache disorders, including likely TTH. These conditions differ in pathophysiology, triggers, and potentially in their socioeconomic distribution [25]. Migraine is typically associated with activation of the trigeminovascular system and neuroinflammatory pathways, whereas TTH is more frequently linked to muscular tension and stress [26, 27]. Moreover, the epidemiological profiles of migraine and TTH may diverge [28, 29], with migraine in women being strongly influenced by hormonal fluctuations, particularly changes in estrogen [30], while TTH appears more closely related to occupational stress, lifestyle factors, and self-perceived health status [31]. Consequently, the non-significant downward pattern observed in SRHD prevalence could reflect differential temporal trajectories across headache subtypes.

The 2020 data point coincided with the COVID-19 pandemic, and data collection took place partly during lockdown periods and the subsequent easing of restrictions. Therefore, the observed decrease in occurrence is likely attributable to behavioral and lifestyle changes experienced during this period. Consequently, conflicting findings have been reported in the literature. A recent meta-analysis determined that headache disorders were two-fold more prevalent in COVID-19 patients than in the healthy population [32]. Furthermore, three different surveys involving 1018 patients in Kuwait [33], 239 in Spain [34], and 133 in Ukraine [35], concluded that the COVID-19 pandemic had an overall negative impact on migraine disease, even though some patients experienced migraine worsening after COVID-19 vaccination [36]. In contrast, a study involving 870 patients reported an improvement in migraine after lockdown, which was associated with several factors, such as working from home, scaling down demanding social lives, and the freedom to choose how to organize your time [37]. Hence, we propose that social determinants may have influenced the prevalence of these conditions during this period [38, 39]. Additionally, changes in healthcare-seeking behavior during the pandemic may have introduced reporting biases. Reduced access to primary care services, fear of contagion, and the reallocation of healthcare resources to COVID-19 management may have contributed to the underdiagnosis and underreporting of non-urgent conditions such as headache [40, 41]. Moreover, the increased reliance on remote consultations could have affected the accuracy of symptom assessment and diagnosis [42, 43]. Simultaneously, heightened stress, disrupted routines, and social isolation may have amplified perceived symptoms in some individuals [34], while others may have deprioritized non-life-threatening complaints [44, 45]. Therefore, the decrease of prevalence observed in 2020 should be interpreted with caution, as it likely reflects a complex mix of behavioral, systemic, and psychosocial factors that are unique to the context of the COVID-19 pandemic.

Furthermore, upon examining the differences between physician-diagnosed and self-reported SRHD prevalences, a trend of diminishing disparity was noted over time. A similar increase in self-reported medical diagnoses of migraine (from 38% in 1989 to 59% in 2018) has been observed in the American population over the past 30 years [46–49]. This decline could stem from an increase in headache-related healthcare visits (mainly primary care) [49] enhanced diagnostic techniques [50–52], heightened awareness of migraine and headache [5, 49, 53], and targeted interventions tailored to particular risk factors [49]. Specifically, in Spain, since the mid-2000s, the Spanish Society of Neurology (Sociedad Española de Neurología, SEN) has promoted awareness campaigns and clinical guidelines on headache diagnosis and management, aiming to improve early detection and appropriate treatment, particularly in primary care settings [54–57]. In addition, several regions have implemented structured headache protocols within primary care, which may have reduced underdiagnosis and improved patient triage [58]. The inclusion of migraine treatments, such as triptans or gepants, in public reimbursement schemes, together with broader improvements in headache recognition and management within primary care, may have contributed to reducing underdiagnosis and improving clinical management [54]. However, it is important to note that newer disease-specific therapies, including monoclonal antibodies targeting CGRP pathways and gepants, have primarily been indicated for chronic or refractory migraine and have only been widely available in Spain during the later years of the study period. While these treatments have substantially improved individual quality of life and disability outcomes, their current population-level penetration is unlikely to be sufficient to meaningfully influence overall prevalence estimates during the timeframe analyzed. Thus, public health initiatives should aim to decrease the observed underdiagnosis of different cephalalgias (which has been broadly recognized) [51, 52, 59].

An analysis based on sex revealed significant disparities, consistently showing higher SRHD prevalence rates (more than double) among women than among men, consistent with findings from previous studies in migraine and TTH patients [19, 22, 60, 61]. Not only is the prevalence higher, but women also have more frequent, longer-lasting, and more severe headaches than men, and consequently, a higher burden of disease [23, 61–63]. The difference in migraine prevalence between sexes is thought to be influenced, at least in part, by variations in ovarian steroid hormones, particularly estrogen and progesterone, although the precise mechanisms remain incompletely understood [61, 64–66]. This theory is supported by the higher prevalence observed during the reproductive years (as observed in our results) [67], in women treated with combined hormonal contraception [68], and during menstruation [69–71]. Meanwhile, pregnancy and post-menopause are associated with improvements in migraine symptoms [72, 73]. Moreover, several authors have found sex-specific structural and functional differences between male and female migraine brains [74, 75], which may be associated with sex hormone modulation [65].

As observed in our analysis, age was a determinant variable influencing SRHD prevalence, with a characteristic pattern where the groups with the highest prevalence were found in the middle-aged groups (between 25 and 54 years) and the lowest in the extreme groups (< 25 and > 54 years). This pattern has been widely reported in migraine patients [19, 22, 23, 61, 76]. The proposed explanation is that this period comprises the reproductive years in which sex hormones have greater fluctuations and more active roles [61, 67]. In addition, it has been suggested that the therapeutic benefits of pharmacological treatment increase with age [77]. Interestingly, according to our data, the decrease in prevalence over time was more pronounced in older age groups.

A noticeable decline in the prevalence of SRHD was noted as socioeconomic status improved, which may be attributed to biological, psychological, and societal factors. Multiple studies have consistently indicated elevated rates of chronic and debilitating pain among individuals of lower socioeconomic status [20, 78, 79], and an equivalent pattern has been observed in migraine and other headache types [80–82]. This disparity was more strongly associated with income above the educational level [80]. In contrast, some studies did not find an association between socioeconomic status (assessed by income or education) and migraine prevalence [83–85], which is consistent with our observations in 2023. According to our data, the reduction in SRHD prevalence was more prominent among individuals from lower social classes, suggesting that socioeconomic disparities may have influenced the temporal dynamics of headache burden.

Notably, an apparent convergence of SRHD prevalence across social classes in 2023 was observed, contrasting with the clearer socioeconomic gradients observed in earlier survey waves. This flattening should be interpreted with caution. While it may reflect a true reduction in disparities, post-pandemic contextual factors such as increased teleworking, screen exposure, and sustained digital workload may have disproportionately affected higher socioeconomic groups, potentially narrowing previously observed differences. In addition, subtle changes in reporting behavior or participation patterns in the post-pandemic period cannot be excluded. Given the cross-sectional design and limited number of time points, it is not possible to determine whether this convergence represents a sustained epidemiological shift or a transient post-pandemic effect.

This study has several limitations that should be acknowledged. First, it relied on self-reported data regarding the presence and diagnosis of migraine or frequent headache, without clinical verification. Accordingly, the findings should be interpreted as reflecting perceived burden rather than formally diagnosed prevalence. Second, the survey design did not allow for differentiation between headache types (e.g., migraine vs. tension-type) or between episodic and chronic forms. Thus, our estimates represent aggregated self-reports and should not be interpreted as subtype-specific prevalence, limiting their comparability with the International Classification of Headache Disorders-3 (ICHD-3)-based epidemiological studies [86]. Furthermore, given the limited number of time points, the Mann-Kendall test had low statistical power, and therefore the observed slope should be interpreted as insufficient evidence to confirm a temporal trend rather than evidence of a true decline. Additionally, given the large number of subgroup and trend analyses conducted, the possibility of type I error due to multiple comparisons cannot be completely excluded. However, we emphasize that our objective was to assess temporal trends in self-perceived headache reporting via standardized public health instruments, not clinical prevalence. As a strength, the use of secondary data from the INE ensures methodological rigor and a large sample size, thus enhancing the reliability of the conclusions drawn. Moreover, as long as the current criteria are met, there is potential to expand this information for ongoing monitoring.

## Conclusions

In summary, analyses of national survey data from 2006 to 2023 indicate a non-significant downward pattern in the prevalence of self-reported headache disorder in the Spanish population aged ≥ 15 years. Changes over time were not homogeneous across demographic and socioeconomic subgroups, with lower prevalence estimates more evident among women, older individuals (≥ 55 years), and those from lower social or educational backgrounds. These findings suggest that the observed changes in headache prevalence may be driven by demographic and socioeconomic factors rather than reflecting a uniform national trend. Further research is needed to identify the determinants underlying these subgroup-specific differences and to determine whether similar patterns occur in other countries. A better understanding of these dynamics will support the design of more targeted and equitable public health strategies for headache prevention and management.

## Supplementary Information

Supplementary Material 1.

## Data Availability

The data that support the findings of this study are available in the following resources available in the public domain: National Statistics Institute of Spain (INE, in the Spanish acronym) at https://www.ine.es/dyngs/INEbase/operacion.htm?c=Estadistica_C&cid=1254736176783&menu=resultados&idp=1254735573175#_tabs-1254736195650 and https://www.ine.es/dyngs/INEbase/es/operacion.htm?c=Estadistica_C&cid=1254736176784&menu=resultados&idp=1254735573175.
