# Supplementary material for: ​​Trends in self-reported headache disorder in Spain between 2006 and 2023: the role of sociodemographic factors
Source: BMC Public Health. 2026 Feb 27;26:1104. doi: 10.1186/s12889-026-26744-3 (PMC13049755; doi:10.1186/s12889-026-26744-3)
Supplement: Supplementary file 1 — Supplementary Material 1. [file 12889_2026_26744_MOESM1_ESM.docx]

**Trends in Self-Reported Headache Disorder in Spain between 2006 and 2023: The Role of Sociodemographic Factors**

**Supplementary Information**

Miguel Á. Huerta^a,b^ MSc, Jose A. Moral-Munoz^c,d,e^* PhD, Masahito Katsuki^f,g^ MD PhD,

Yasuhiko Matsumori^h^ MD PhD, Alejandro Salazar^d,e,i^ PhD

^a^ Department of Pharmacology and Neurosciences Institute (Biomedical Research Center), University of Granada.

^b^ Biosanitary Research Institute ibs.GRANADA, Granada, Spain.

^c^ Department of Nursing and Physiotherapy, University of Cadiz, Cadiz, Spain.

^d^ Biomedical Research and Innovation Institute of Cadiz (INiBICA), Cadiz, Spain.

^e^ Observatory of Pain, Grünenthal Foundation-University of Cadiz, Cadiz, Spain.

^f^ Insight Science Foundation Ireland Research Centre for Data Analytics, School of Human and Health Performance, Dublin City University, Dublin, Ireland.

^g^ Physical Education and Health Center, Nagaoka University of Technology, Niigata, Japan.

^h^ Sendai Neurology and Headache Clinic, Sendai, Miyagi, Japan.

^i^ Department of Statistics and Operational Research, University of Cadiz, Cadiz, Spain.

***Corresponding author:** Jose A. Moral-Munoz. Address: Av. Ana de Viya, 52, 11009 Cádiz, Cádiz (Spain). Email: [joseantonio.moral@uca.es](mailto:joseantonio.moral@uca.es)

**
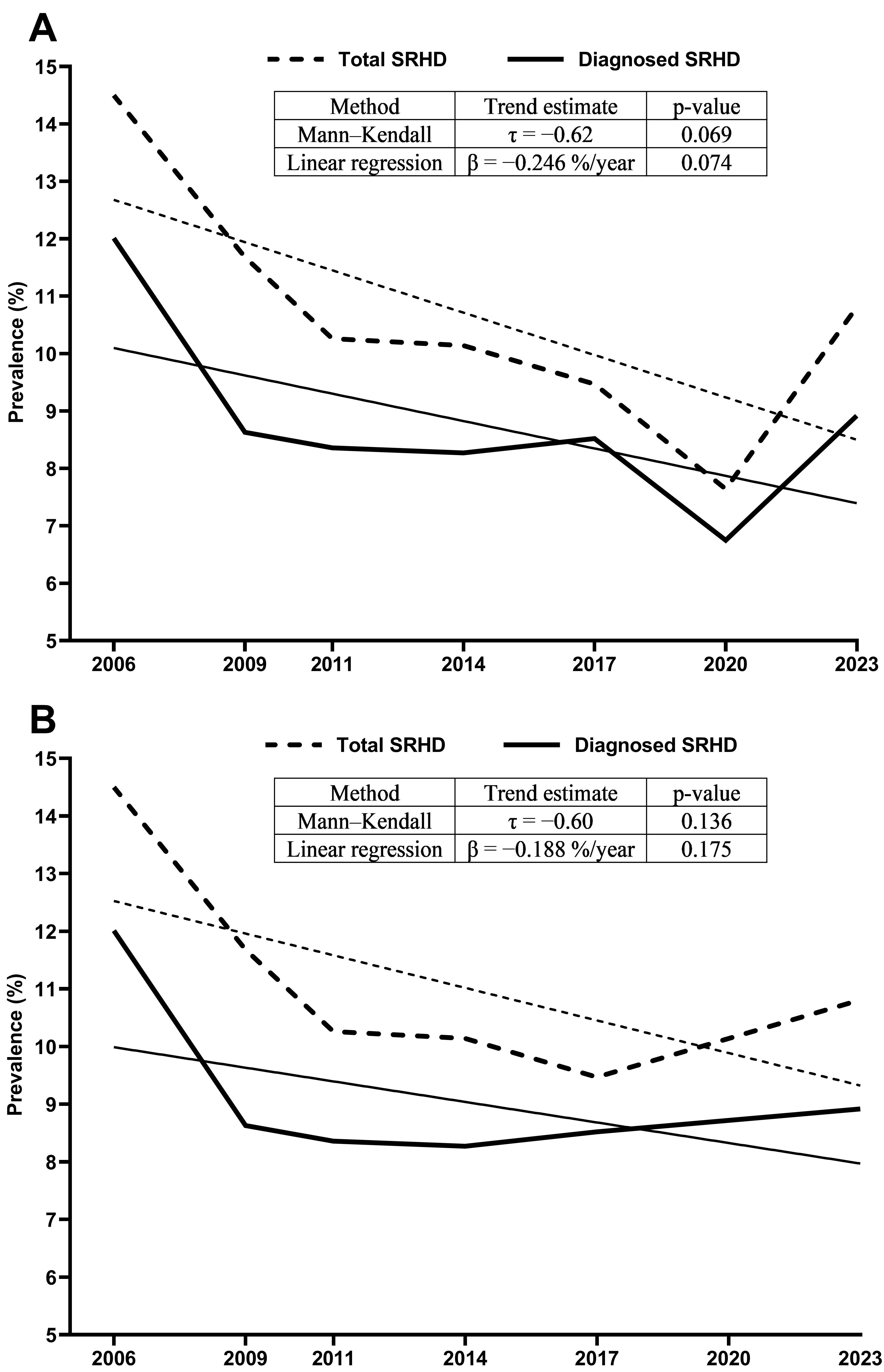
**

**Figure S1. Sensitivity analysis of temporal trends in self-reported headache disorder prevalence in Spain.** Temporal evolution of SRHD prevalence in the Spanish population aged ≥15 years. Panel A shows trend analyses including the 2020 survey, collected during the COVID-19 pandemic, while Panel B shows analyses excluding this data point. Dashed lines represent total self-reported SRHD prevalence, and solid lines represent physician-diagnosed SRHD. Abbreviation: Self-reported headache disorder, SRHD.
